# Efficiency of an Online Health-Promotion Program in Individuals with At-Risk Mental State during the COVID-19 Pandemic

**DOI:** 10.3390/ijerph182211875

**Published:** 2021-11-12

**Authors:** Ching-Lun Tsai, Cheng-Hao Tu, Jui-Cheng Chen, Hsien-Yuan Lane, Wei-Fen Ma

**Affiliations:** 1 Department of Public Health (in Epidemiology and Preventive Medicine), China Medical University, No. 100, Sec. 1, Jingmao Rd., Beitun Dist., Taichung 406040, Taiwan; chris.tsai70@gmail.com; 2 Graduate Institute of Acupuncture Science, China Medical University, No. 91 Hsueh-Shih Road, Taichung 404333, Taiwan; lordowen@mail.cmu.edu.tw; 3 Neuroscience Laboratory, Department of Neurology, China Medical University Hospital, No. 2, Yude Road, North District, Taichung 40447, Taiwan; andrewtw717@gmail.com; 4 School of Medicine, China Medical University, No. 91 Hsueh-Shih Road, Taichung 404333, Taiwan; 5 Department of Neurology, China Medical University, No. 199, Sec. 1, Xinglong Rd., Hsinchu County 302056, Taiwan; 6 Graduate Institute of Biomedical Sciences, China Medical University, No. 91 Hsueh-Shih Road, Taichung 404333, Taiwan; 7 Department of Psychiatry, China Medical University Hospital, No. 2, Yude Road, North District, Taichung 404332, Taiwan; 8 Department of Psychology, College of Medical and Health Sciences, Asia University, No. 500, Lioufeng Rd., Wufeng, Taichung 41354, Taiwan; 9 PhD Program for Health Science and Industry and School of Nursing, China Medical University, No. 100, Sec. 1, Jingmao Rd., Beitun Dist., Taichung 406040, Taiwan; 10 Department of Nursing, China Medical University Hospital, No. 2, Yude Road, North District, Taichung 404332, Taiwan

**Keywords:** at-risk mental state, online health-promotion program, social media, COVID-19

## Abstract

Mental health issues caused by the COVID-19 pandemic greatly impact people’s daily lives. Individuals with an at-risk mental state are more vulnerable to mental health issues, and these may lead to onset of full psychotic illnesses. This study aimed to develop and evaluate an online health-promotion program for physical and mental health of the individuals with at-risk mental state during the COVID-19 pandemic. A single group study with pre- and post-tests was conducted in 39 young adults with at-risk mental state. The participants were provided with the online health-promotion program after completing the pretest. Via social media, the online counseling program released one topic of material (about 15–20 min) every two weeks and provided interactive counseling for specific personal health needs on the platform. Study questionnaires, physiological examination, and blood serum examination were completed at both pre- and post-tests. The participants showed significant improvements in mental risk, anxiety, and physical activity after participating in the program. Furthermore, those who did not complete the program had significantly more severe negative symptoms. These results imply that the online health-promotion program is effective and accessible under certain barriers such as the COVID-19 pandemic, but not for individuals with higher risk of more negative mental health symptoms.

## 1. Introduction

The coronavirus disease 2019 (COVID-19) is a crisis posing tremendous challenges for many different societies, countries, and their respective cultures worldwide [1]. In 2020, it had reached pandemic level and caused approximately 170 million confirmed cases and 3 million deaths globally [2]. The anxiety, fear, stress, and distress caused by the pandemic and quarantine significantly changed the daily lives of many people. Facing new scenarios of home-schooling; lacking physical contact with family, friends, and colleagues; having temporary unemployment, etc. all caused huge impacts on people’s physical and mental health [1,3].

Several studies had suggested that the COVID-19 pandemic was associated with psychiatric symptoms of depression [4], anxiety [5], psychological distress [6], and PTSD symptoms [4,7] in the general population of adults and children. Some of the symptoms were present in up to 28.8% of adults [5]. In addition, many healthcare workers reported high levels of stress, distress, burnout, sleep problems, anxiety, and depression [8,9].

The individual with at-risk mental state (ARMS) may be more vulnerable due to having a higher level of perceived stress and anxiety than the general public [10,11]. Although their diseases have not yet been fully developed, the symptoms have already affected their life function. Those individuals may have a risk of developing their first episode of psychosis within two years [12,13] and up to one third of them may progress into full psychotic illness within three years [13,14,15]. Appling early intervention in this population is highly recommended.

In chronic diseases, health promotion is considered as one of the vital strategies for health improvement and symptom management [16]. The WHO (2004) recognized health promotion as the activity that enables people to adopt and maintain healthy lifestyles, and creates favorable healthy environments and living conditions [17]. It includes the activities that actualize personal health potential and increase the level of wellbeing [18]. A previous study has demonstrated the effectiveness of health promotion in individuals with ARMS in the aspects of improving mental risk, anxiety level, health-promoting lifestyles, quality of life, physical exercise, and physical health [19].

In the present technological era, the health services on online platforms or telehealth have been experiencing increasing popularity [20]. This refers to the use of computers, mobile devices, and the internet to communicate and exchange information [21]. It can bring more opportunity for people to gain better access to and quality of healthcare services, when the access to healthcare meets an obstacle such as the COVID-19 pandemic [3,22,23].

The effectiveness of the online interventions has already been found to be effective in the prevention or treatment of depression [24,25], anxiety [25], and social phobia [26]. A previous study on web- and mobile app-based mental health-promotion intervention also demonstrated that the people who attended more online sessions showed significantly greater improvements in mental health, vitality, depression, and life satisfaction compared to the ones who attended less sessions [27]. This study aimed to evaluate the effectiveness of the Online Health-Promotion Program (OHPP) on physical and mental health of the young Taiwanese adults with ARMS under the COVID-19 pandemic.

## 2. Materials and Methods

### 2.1. Study Design and Data Collection

A quasi-experimental single group was for this study design. A purposive sampling group of individuals with ARMS was enrolled to receive pre- and post-tests from March 2020 to May 2021. The participants were provided with the OHPP program after completing the pretest. The assessments were finished one week before and after the program to evaluate its effectiveness.

### 2.2. Participants

The study subjects were referred by psychiatrists from psychiatric clinics. They were enrolled into this study if they (1) were 13–45 year old males or females; (2) were under high mental risk, which included one of the following: total Scale of Prodromal Symptoms [SOPS] score ≧ 20 [28], total Chinese Version of Schizotypal Personality Questionnaire-Brief (CSPQ-B) score ≧ 17 [29], or total Chinese Mandarin State and Trait Anxiety Inventory Form Y (CMSTAI-Y) score ≧ 60 [11,30]; (3) were physically capable of doing exercise; and (4) agreed to participate in the study and provided written informed consent after complete description of the study. For the subjects < 20 years old, a parent also cosigned the written informed consent. The exclusion criteria included: (1) premorbid IQ < 70; (2) current abuse of alcohol or drugs; and (3) DSM-5 criteria for schizophrenia, mood disorders, or other psychotic disorders.

### 2.3. Online Health-Promotion Program

The goals of the Online Health-Promotion Program (OHPP) were to reduce mental risk, stabilize mood, and improve both physical and psychological well-being through online counseling. The main topics for the online counseling were modified from the HASL program as conducted in the previous studies, which included exercise [31], stress management [11], and health responsibility [19]. The online counseling materials included a total of three topics and were released on social media, in the manner of one topic every two weeks. Approximately 15–20 min were needed to complete each topic. The detailed procedures of OHPP are shown in Table 1.

The online counseling focused on using the existing and most commonly used social media to promote collaboration between the participants and the researchers in order to achieve better health outcomes. This study chose to use LINE as the social media online platform because it has been preferred by 84% of Taiwanese internet users aged 16–64 since 2019 [32].

LINE was implemented on mobile devices or computer in the form of Apps. The researcher used the research account to add the participants on LINE during the pretest. The sharing of messages, photos, and videos on the LINE App with the participants was under appropriate privacy settings of confidentiality.

The online counseling aspect answered any questions in a quick direct message; enhanced the relationship between the participant and the researcher; tracked the participant’s individual progress of symptom management, exercise plan, and life events; accessed stress adjustment and emotional expression; provided physical and mental health knowledge and advice; advised seeking medical help if necessary; and reduced the possible anxiety and stigma caused by in-person counseling.

In addition, a wearable fitness tracker was also provided to the participants during the pretest. The wearable fitness tracker had standard fitness functions such as recording all-day activities, sports, and sleep; detecting heart rate; and calculating walking distance and calorie consumption. It served as an aid for analyzing, interpreting, and evaluating the data in a real-time manner for the success of the individual exercise plan, sleep quality, and symptom improvement. It also acted as a reminder for when participants had taken <1000 steps at noon time daily for exercise encouragement.

### 2.4. Instruments

This study had seven assessment tools, including (1) demographic inventory, (2) Chinese Version of Schizotypal Personality Questionnaire-Brief (CSPQ-B) [29], (3) Brief Self-Report Questionnaire for Screening Putative Pre-Psychotic States (BQSPS) [33], (4) Scale of Prodromal Symptoms (SOPS) [28], (5) Chinese Mandarin State and Trait Anxiety Inventory Form Y (CMSTAI-Y) [11,30], (6) 3-Month Physical Activity Checklist (3MPAC) [34], and (7) physiological index and blood serum examination.

#### 2.4.1. Demographic Inventory

Information on age, education years, gender, marital status, religious belief, previous experiences seeking help for mental health, and family members with history of mental illness, was obtained from the participant’s self-report.

#### 2.4.2. Chinese Version of Schizotypal Personality Questionnaire-Brief (CSPQ-B)

CSPQ-B is a Chinese version of Schizotypal Personality Questionnaire-Brief (SPQ-B) [29]. It is a 22-item questionnaire developed by Raine and Benishay in 1995 [35]. The questionnaire contains eight questions related to cognitive–perceptual factors, eight questions on interpersonal factors, and another six questions which were related to being disorganized. Higher scores indicate a greater degree of mental deficit. The optimal cutoff score was 17, obtained from 618 undergraduate students, and the sensitivity and specificity were 80.0% and 85.9%, respectively [29]. CSPQ-B had also been used in 149 ketamine-dependent patients aged 18–65 [36], 52 healthcare-related undergraduates with ARMS [11], and 92 young adults aged 19–26 with ARMS [19].

#### 2.4.3. Brief Self-Report Questionnaire for Screening Putative Pre-Psychotic States (BQSPS)

BQSPS is a 15-item, self-report scale for identifying the early and broadly defined pre-psychotic states using a dichotomous Yes/No questionnaire [33]. BQSPS addresses four symptomatic categories, including five interpersonal difficulty/social anxiety symptoms, three self-depreciating descriptions, three negative symptoms, and four subthreshold psychotic-like experiences [33]. The questionnaire has two cutoff points for being putatively pre-psychotic: (1) answering yes more than or equal to eight times, (2) answering yes in between three to seven times plus answering yes to any of the following three items, “I cannot deal with the pressures associated with crowds” (Item 1), “I feel I cannot get close to people” (Item 2), “Do you hear some sounds, voices, or calls of your name when nobody is around you?” (Item 15) [33]. The estimated averaged sensitivity and specificity of BQSPS were 0.736 and 0.679, respectively [33].

#### 2.4.4. Scale of Prodromal Symptoms (SOPS)

SOPS is a 19-item self-report questionnaire developed to identify individuals experiencing early signs of psychosis [37]. It consists of four subscales: five items in positive symptoms, six items in negative symptoms, four items in disorganization, and four items in general symptoms [37]. Higher scores indicate more severe psychiatric symptoms. SOPS had Cronbach’s alpha indices were 0.880 in the recruitment phase and 0.952 one year later. Furthermore, the negative symptoms were found to have the best specificity (95.5%) and sensitivity (100%) [38].

#### 2.4.5. Chinese Mandarin State and Trait Anxiety Inventory Form Y (CMSTAI-Y)

CMSTAI-Y is a Chinese version of State and Trait Anxiety Inventory Form Y (STAI-Y) [30]. It is a 4-point Likert scale with 20 items, each with state anxiety and trait anxiety. Those who score over 60 points are considered to be suffering from high levels of anxiety [39]. CMSTAI-Y, tested in 306 Taiwanese adults with anxiety disorders, showed test–retest reliabilities of 0.76–0.91 over two weeks; its Cronbach’s α values for internal consistency of State and Trait Anxiety were 0.91 and 0.92, respectively [30]. CMSTAI-Y has been also applied among 19–45 year-old college nursing students in Taiwan [40], 239 adults aged 20–60 with higher anxiety levels [41], 86 patients with anxiety disorders [31], and 52 healthcare-related undergraduates with ARMS [11].

#### 2.4.6. 3-Month Physical Activity Checklist (3MPAC)

The 3MPAC, an 18-item, self-report scale, measures physical activity levels in the past three months for adults with mental disorders [34]. The criterion for validity testing with a 7-Day Physical Activity Recall interview was r = 0.47 for light, r = 0.64 for moderate, and r = 0.73 for heavy exercises and cross-sample testing was χ2 = 21.98, *p* < 0.000 in the groups of 98 schizophrenia adults, 153 adults with anxiety disorders, and 22 bipolar adults [34]. The test–retest reliability of light, moderate, and heavy exercises were 0.71 to 0.86 [34]. The 3MPAC has been also used in 92 young adults aged 19–26 with ARMS [19].

#### 2.4.7. Physiological Index and Blood Serum Examination

The body mass index (BMI), systolic blood pressure (BP), diastolic BP, and waist/hip ratio were all measured by noninvasive approaches and calculated accordingly. The glucose (AC), triglyceride, and HDL were measured by blood testing and collected in the lab of the study site’s medical center.

### 2.5. Ethical Considerations

The procedure of this study was reviewed and approved by the ethical review board of the study site. The informed consent was signed by all the participants. For subjects <20 years old, their parents also cosigned the written informed consent. The participants’ names were confidential throughout the study. The data were not used by a third party. Participants’ rights of medical treatment were not influenced after withdrawal.

### 2.6. Data Analysis

SPSS for Windows 22.0 software was used for data analysis. The descriptive statistics consisted of percentages, mean values, standard deviations (SD). The inferential statistical analysis consisted of a two-sample independent *t*-test, paired *t*-test, and McNemar’s test [42]. The statistically significant level was set at less than 0.05.

## 3. Results

### 3.1. Demographic Characteristics

There were 39 participants with ARMS recruited in the study. The mean age was 25.74 years (SD = 7.09) and mean education in years year was 14.21 (SD = 2.04). Most of the participants were female 24 (61.5%), and their marital status for single, married, and divorced were 32 (82.1%), 4 (10.3%), and 3 (7.7%), respectively. Almost half of them reported to be religious (19, 48.7%). Among the 39 participants, 20 (51.3%) had previous experience seeking help for their mental health, and 15 (38.5%) had family members with history of mental illness.

Among the 39 participants, 24 (61.5%) completed all three topics of the OHPP online counseling materials. In comparison with the ones who decided not to continue, those who finished the OHPP had significantly higher BMI (t = 2.26, *p* = 0.03), higher systolic BP (t = 2.13, *p* = 0.04), lower scores for total putative pre-psychotic states (t = −2.28, *p* = 0.03), lower interpersonal difficulty/social anxiety symptoms (t = −2.08, *p* = 0.04), lower negative symptoms (t = −3.05, *p* = 0.00), lower total prodromal symptoms (t = −2.96, *p* = 0.01), and lower negative symptoms of the prodromal symptoms (t = −3.35, *p* = 0.00). The differences in study variables between those who completed the OHPP and those who did not are shown in Table 2.

### 3.2. The Effectiveness of the OHPP

For those who completed the OHPP, they showed significant improvements in state anxiety (t = 4.72, *p* = 0.00), trait anxiety (t = 4.79, *p* = 0.00), total schizotypal personality (t = 2.14, *p* = 0.04), disorganized thoughts (t = 2.58, *p* = 0.02), total putative pre-psychotic states (t = 2.45, *p* = 0.02), and all dimensions of the prodromal symptoms. In addition, the number of people suffered from trait anxiety was reduced significantly by 25% (*p* = 0.03). The results of the changes in anxiety and mental risks between pre- and post-test are shown in Table 3.

As for the physical and physiological aspect of the assessments, the physical assessments and serum assessments did not show significant changes. However, the amount of moderate aerobic exercise was increased significantly (t = −2.23, *p* = 0.04) with double the number of people doing moderate aerobic exercise for over 150 min per week (*n* = 6, 25.0% in T0; *n* = 12, 50.0% in T1; *p* = 0.03). The differences in physical and physiological variables between pre- and post-test are shown in Table 4.

## 4. Discussion

The online health-promotion program of this study yielded significant improvements in overall mental risks, anxiety, and physical activity level for young Taiwanese people with ARMS during the COVID-19 pandemic. The result was very similar to that of the previous study using the Health-Awareness-Strengthening Lifestyle (HASL) program [19]. This finding suggests that the online version of this health-promotion program not only serves its original purpose of increasing the physical and mental health of young persons with ARMS but also increases the accessibility by using an online platform during certain barriers such as the COVID-19 pandemic [3,22,23].

Most of the current online mental health interventions were designed for the symptoms of depression [24,25,43,44,45], mood [46], anxiety [25,43,44], and stress [44,47,48] in general populations. As for psychosis-related symptoms, a pilot study in 10 youth with first-episode psychosis using two-month online intervention showed significant improvement in social anxiety [49]. Another study by Ludwig et al. (2020) on 26 participants with schizophrenia spectrum disorder using 12-week, online social-media platforms showed improvements in psychosis-related symptoms [50]. In addition, Peck et al. (2020) created a protocol for a web-based peer-support program using 14 videos on the topics of my journey, my identity, self-care, life, connections, and mental health for young people experiencing psychosis [51]; however, future research was still required for assessing the effectiveness of the program. Compared to the programs for other mental health symptoms, those for psychosis have been limited, and there are even fewer for ARMS. The results of this study showed that OHPP has beneficial effects on mental risks, such as schizotypal personality, disorganized thoughts, putative pre-psychotic states, prodromal symptoms, as well as anxiety symptoms for young people with ARMS.

In most health-promotion programs, including some online programs [52,53], stress management is one of the most common and effective strategies for improving mental health, depression, anxiety, stress, sleeping problems, etc. Physical activity is another element frequently implemented in health-promotion programs for physical wellbeing, stress management, nonpharmacological disease prevention, etc. [43,53,54,55]. Health responsibility is a dimension of health promotion that is often applied by researchers. For instance, a mobile health intervention for mental health promotion based on wellbeing, validated self-help exercises, brief tips, self-monitoring, and personalized feedback showed significantly better results for positive mental health, depression, and anxiety symptomatology [43]. The program created by Peck et al. (2020), aforementioned, also implemented some elements of health responsibility [51]. The current study also adopted the strategies of exercise, stress management, and health responsibility to increase the mental wellbeing of the young individuals with ARMS.

Though online health-promotion programs implemented different online methodologies, similar features, such as educational videos [51,52,53,54], text messages [43,52], social forums [53], and external exercise links [43,54], were adopted. This study utilized educational videos for conveying health information, and social media for interaction. In addition, a wearable fitness tracker was applied for daily exercise encouragement.

This study had several limitations. First, a control group was not recruited due to ethical considerations. Another limitation was the small sample size; one reason for this was the unwillingness of people to visit a medical center for conducting study questionnaires, physiological examinations, and blood serum examinations during pre- and post-test. Future research should take this concern into account. Lastly, the applicability of OHPP in other social and cultural settings also needs to be further assessed.

It also deserves attention that those participants who did not finish all three online sessions scored significantly higher in putative pre-psychotic states, interpersonal difficulty, social anxiety symptoms, negative symptoms, and prodromal symptoms than those who completed all three online sessions. According to DSM-5, negative symptoms are clustered into two main domains: diminished emotional expression (i.e., poverty of speech, flat affect, few gestures, etc.) and avolition (i.e., apathy, anhedonia, lack of interest, social withdrawal, etc.) [56,57]. The symptoms of avolition may affect compliance in the online program continuation. An addition of a few in-person counterpart sessions is recommended for future study, in order to increase more therapeutic relationships, supports, and encouragements. Furthermore, we also found that the participants who completed all three online sessions also had significantly higher values in BMI and systolic BP initially in the pretest than those who did not finished.

## 5. Conclusions

This study developed an online version of a health-promotion program to reduce mental risk, stabilize mood, improve both physical and psychological wellbeing, and try to delay or avoid the onset of psychosis through online counseling. The results revealed that the OHPP significantly improved mental risk, anxiety, and physical activity. These findings can be the basis for the development of online health-promotion programs for individuals with ARMS under certain barriers such as the COVID-19 pandemic.

## Figures and Tables

**Table 1:** The detailed procedures of Online Health-Promotion Program.

Session Topic	Definitions	Counseling Strategies	Online Methods
Exercise	Engaging in sports and leisure activities	Establishing role model Providing mechanisms linking exercise and stress management	Video
		Encouraging sharing of previous sports experience	Social media interaction
		Exercise reminder and encouragement	Wearable fitness tracker
Stress Management	Using relaxation techniques for stress management	Encouraging the use of regular moderate aerobic exercise as the stress management ability Training of crisis management and response capabilities	Video
		Advising time management and planning	Social media interaction
Health Responsibility	Paying attention to health condition Seeking professional assistance if needed Attending health lesson	Establishing a health responsibility role model	Video
		Encouraging sharing of health responsibility issues	Social media interaction

**Table 2:** The differences in study variables during pretest.

	Total (*n* = 39)	Completed (*n* = 24)	Drop out (*n* = 15)	*t*	*p*
	Mean (SD)	Mean (SD)	Mean (SD)		
Physical Assessments					
BMI	23.26 (5.11)	24.65 (5.45)	21.04 (3.67)	2.26	0.03
Systolic BP	111.46 (15.98)	115.58 (15.94)	104.87(14.15)	2.13	0.04
Diastolic BP	74.79 (9.67)	76.67 (9.72)	71.80 (9.13)	1.56	0.13
Waist/Hip Ratio	0.83 (0.07)	0.84 (0.08)	0.80 (0.06)	1.62	0.11
Serum Assessments					
Glucose (AC)	91.92 (11.30)	93.55 (10.45)	89.87 (12.77)	0.90	0.37
Triglyceride	104.81 (95.70)	112.77 (114.48)	82.64 (46.33)	1.10	0.28
HDL	55.68 (15.23)	55.09 (16.14)	57.89 (13.67)	−0.72	0.48
Physical Activities					
Moderate Aerobic Exercise	110.66 (179.40)	95.39 (146.20)	129.78 (220.45)	−0.52	0.61
Anxiety					
State Anxiety	52.21 (9.96)	50.50 (10.65)	54.93 (8.35)	−1.37	0.18
Trait Anxiety	62.15 (9.53)	60.00 (9.59)	65.60 (8.64)	−1.84	0.07
Schizotypal Personality					
Cognitive–Perceptual Deficits	5.03 (1.88)	4.96 (1.85)	5.13 (2.00)	−0.28	0.78
Interpersonal Deficits	6.15 (2.37)	5.83 (2.51)	6.67 (2.09)	−1.07	0.29
Disorganization	3.49 (1.70)	3.21 (1.64)	3.93 (1.75)	−1.31	0.20
CSPQ-B Total	14.67 (5.14)	14.00 (5.00)	15.73 (5.36)	−1.02	0.31
Putative Pre-psychotic States					
Interpersonal Difficulty/Social Anxiety Symptoms	3.72 (1.30)	3.42 (1.41)	4.20 (0.94)	−2.08	0.04
Self-Depreciating Descriptions	2.23 (0.96)	2.13 (0.95)	2.40 (0.99)	−0.87	0.39
Negative Symptoms	1.85 (1.06)	1.50 (1.10)	2.40 (0.74)	−3.05	0.00
Subthreshold Psychotic-Like Experiences	2.85 (1.06)	2.67 (1.09)	3.13 (0.99)	−1.35	0.19
BQSPS Total	10.64 (3.41)	9.71 (3.38)	12.13 (2.97)	−2.28	0.03
Prodromal Symptoms					
Positive Symptoms	7.11 (3.63)	6.73 (3.19)	7.60 (4.31)	−0.78	0.44
Negative Symptoms	10.97 (5.75)	8.50 (4.98)	14.27 (5.24)	−3.35	0.00
Disorganization Symptoms	3.84 (2.59)	3.73 (2.29)	4.07 (3.01)	−0.48	0.63
General Symptoms	8.11 (2.86)	7.50 (2.89)	8.93 (2.69)	−1.53	0.13
SOPS Total	30.03 (9.41)	26.45 (6.34)	34.87 (11.30)	−2.96	0.01

Note. BMI = body mass index, BP = blood pressure, AC = before meals, HDL = high-density lipoprotein, CSPQ-B = Chinese Version of Schizotypal Personality Questionnaire-Brief, BQSPS = Brief Self-Report Questionnaire for Screening Putative Pre-Psychotic States, SOPS = Scale of Prodromal Symptoms.

**Table 3:** The differences in anxiety and mental risks between pre- and post-test.

	Pre-Test (*n* = 24)	Post-Test (*n* = 24)	Paired*-t*	*p*
	Mean (SD)	Mean (SD)		
Anxiety				
State Anxiety	50.50 (10.65)	41.92 (8.25)	4.72	0.00
State Anxiety ≧ 60	4 (16.7%)	0 (0.0%)		^b^
Trait Anxiety	60.00 (9.59)	56.25 (10.21)	4.79	0.00
Trait Anxiety ≧ 60	14 (58.3%)	8 (33.3%)		0.03 ^a^
Schizotypal Personality				
Cognitive-Perceptual Deficits	4.96 (1.85)	4.38 (2.26)	1.33	0.20
Interpersonal Deficits	5.83 (2.51)	5.42 (2.87)	1.36	0.19
Disorganization	3.21 (1.64)	2.46 (1.32)	2.58	0.02
CSPQ-B Total	14.00 (5.00)	12.25 (5.74)	2.14	0.04
CSPQB ≧ 17	8 (33.3%)	6 (25.0%)		0.69 ^a^
Putative Pre-psychotic States				
Interpersonal Difficulty/Social Anxiety Symptoms	3.42 (1.41)	3.17 (1.74)	1.10	0.28
Self-Depreciating Descriptions	2.13 (0.95)	1.92 (1.06)	1.04	0.31
Negative Symptoms	1.50 (1.10)	1.17 (1.09)	1.62	0.12
Subthreshold Psychotic-Like Experiences	2.67 (1.09)	2.25 (1.33)	1.93	0.07
BQSPS Total	9.71 (3.38)	8.50 (3.93)	2.45	0.02
BQSPS ≧ 8	21 (87.5%)	19 (79.2%)		0.63 ^a^
Prodromal Symptoms				
Positive Symptoms	6.73 (3.19)	4.68 (3.48)	4.00	0.00
Negative Symptoms	8.50 (4.98)	6.50 (4.90)	2.71	0.01
Disorganization Symptoms	3.73 (2.29)	2.73 (1.49)	2.13	0.05
General Symptoms	7.50 (2.89)	5.50 (2.65)	3.01	0.01
SOPS Total	26.45 (6.34)	19.41 (8.20)	3.98	0.00
SOPS ≧ 20	24 (100%)	13 (54.2%)		^b^

Note. CSPQ-B = Chinese Version of Schizotypal Personality Questionnaire-Brief, BQSPS = Brief Self-Report Questionnaire for Screening Putative Pre-Psychotic States, SOPS = Scale of Prodromal Symptoms, ^a^ McNemar’s test, ^b^ Sample size = 0 or 100, cannot be tested by McNemar’s test.

**Table 4:** Differences in physical and physiological variables between pre- and post-test.

	Pre-Test (*n* = 24)	Post-Test (*n* = 24)	Paired*-t*	*p*
	Mean (SD)	Mean (SD)		
Physical Assessments				
BMI	24.65 (5.45)	24.68 (5.46)	−0.16	0.87
Systolic BP	115.58 (15.94)	117.58 (19.73)	−0.73	0.48
Diastolic BP	76.67 (9.72)	75.54 (12.23)	0.56	0.58
Waist/Hip Ratio	0.84 (0.08)	0.84 (0.08)	−0.27	0.79
Serum Assessments				
Glucose (AC)	93.55 (10.45)	94.00 (13.93)	−0.12	0.91
Triglyceride	112.77 (114.48)	88.45 (51.15)	1.14	0.27
HDL	55.09 (16.14)	54.93 (14.48)	0.09	0.93
Metabolic Syndrome	9 (37.5%)	6 (25.0%)	23.19	0.45
Physical Activities				
Moderate Aerobic Exercise	95.39 (146.20)	210.12 (337.56)	−2.23	0.04
Moderate Aerobic Exercise ≧ 150	6 (25.0%)	12 (50.0%)		0.03 ^a^

Note. BMI = body mass index, BP = blood pressure, AC = before meals, HDL = high-density lipoprotein, ^a^ McNemar’s test.

## Data Availability

These study data are identified participant data. The data that support the findings of this study are available beginning 12 months and ending 36 months following the article publication from the corresponding author, W-FM, upon reasonable request at lhdaisy@mail.cmu.edu.tw.
